# Prognostic value of the S100 calcium-binding protein family members in hepatocellular carcinoma

**DOI:** 10.1042/BSR20222523

**Published:** 2023-07-06

**Authors:** Ran Wei, Ou Qi Feng, Yao Ze Hui, Xiaohui Huang, Li Sheng Ping

**Affiliations:** 1 Sun Yat-sen University Cancer Center, State Key Laboratory of Oncology in South China, Collaborative Innovation Center for Cancer Medicine, Guangzhou, Guangdong, P.R. China; 2 General Surgical Laboratory, The First Affiliated Hospital of Sun Yat-Sen University, Guangzhou, Guangdong, P.R. China

**Keywords:** hepatocellular carcinoma, risk score model, S100 protein family, TCGA

## Abstract

Hepatocellular carcinoma (HCC) remains a crucial public health problem around the world, and the outlook remains bleak. More accurate prediction models are urgently needed because of the great heterogeneity of HCC. The S100 protein family contains over 20 differentially expressed members, which are commonly dysregulated in cancers. In the present study, we analyzed the expression profile of S100 family members in patients with HCC based on the TCGA database. A novel prognostic risk score model, based on S100 family members, was developed using the least absolute shrinkage and selection operator regression algorithm, to analyze the clinical outcome. Our prediction model showed a powerful predictive value (1-year AUC: 0.738; 3-year AUC: 0.746; 5-year AUC: 0.813), while two former prediction models had less excellent performances than ours. And the S100 family members-based subtypes reveal the heterogeneity in many aspects, including gene mutations, phenotypic traits, tumor immune infiltration, and predictive therapeutic efficacy. We further investigated the role of S100A9, one member with the highest coefficient in the risk score model, which was mainly expressed in para-tumoral tissues. Using the Single-Sample Gene Set Enrichment Analysis algorithm and immunofluorescence staining of tumor tissue sections, we found that S100A9 may be associated with macrophages. These findings provide a new potential risk score model for HCC and support further study of S100 family members in patients, especially S100A9.

## Introduction

Hepatocellular carcinoma (HCC) constitutes nearly 80% of primary liver cancers and is the sixth most common diagnosed cancer and third leading cause of cancer death in 2020 [1,2]. Despite the advancement in treatment options including surgery, radiation, chemotherapy, and molecular therapies, prognosis of HCC is still poor owing to the presence of locally advanced or widely metastatic tumors [3–5]. Thus, novel therapeutic biomarkers and prognostic indicators are paramount for HCC [6].

S100 protein family members (S100s) are a group of calcium binding proteins that regulate calcium homeostasis and other fundamental cellular processes, including proliferation, apoptosis, differentiation, and inflammation [7]. At present, there are more than twenty human S100 proteins have been identified (S100A1-S100A14, S100A7A, S100A16, S100B, S100P, S100G, S100Z, and TCHHL1) [8]. It has been reported that the expression of S100s are commonly dysregulated in human malignancies, such as lung [9], breast [10], colorectal [11], and pancreatic cancer [12], as well as HCC [13]. Moreover, the higher expression of S100A1 [14], S100A4 [15], S100A9 [16], S100A10 [17], S100A14 [18], and S100P [19] has been shown to be associated with shorter survival among HCC patients. Therefore, S100s proteins may serve as biological markers and therapeutic targets for HCC because of their key roles in tumorigenesis and cancer progression. Tumor microenvironment (TME) is composed of cancer cells and non-cancerous cells, which plays a vital role in HCC progression and greatly impacts immunotherapeutic outcomes [20]. It is still unclear how S100s affect the immune microenvironment in HCC.

With the progress of next-generation sequencing, molecular subtyping of HCC has been emerging. HCC has been classified according to transcriptional data, tumor genomes and epigenomes [21]. However, the aforementioned methods still have some limitations. The aim of the present study was to construct a prognostic model based on S100 gene family expression in HCC using least absolute shrinkage and selection operator (LASSO) method. LASSO is a kind of compression estimation that utilizes attribute selection and regularization to improve the predictability and interpretability of the final statistical model. By constructing a penalty function, it compresses some coefficients and allows some coefficients to become zero, which implies that the advantages of subset shrinkage are retained. In our study, LASSO regression was performed to simplify and regularize the S100 family members-based model for HCC patients. The new S100 family members-based risk score model of HCC exhibited substantial heterogeneity in many aspects, including prognosis, genetic mutations, phenotypic traits (measured as scores in TCGA [22]), predictive therapeutic efficacy and the abundance of infiltrating immune cell. The present study provides a comprehensive analysis of S100 family members and suggests an essential function of them in liver cancer development.

## Methods

### Data processing

From the Genomic Data Commons (GDC) data portal (https://portal.gdc.com, accessed on 1 March 2022), we downloaded RNA-sequencing expression profiles and clinical information for 371 HCC patients (one patient was excluded because of missing information) (Supplementary Table S1) and 50 para-tumor tissues (called ‘solid tissue normal’ in TCGA dataset) that are taken from normal tissues near the tumor. In addition, transcriptomics data with clinical features of 232 HCC patients (including 240 primary tumor samples and 197 normal samples) in LIRI-JP project were downloaded from the ICGC database (https://dcc.icgc.org/releases/current/Projects, accessed on March 1, 2022) (Supplementary Table S2). For proteomics data, 165 paired HCC and non-tumor normal cases were obtained from Clinical Proteomic Tumor Analysis Consortium (CPTAC, https://proteomics.cancer.gov/programs/cptac, accessed on March 1, 2022). All tumor samples were pathologically diagnosed as hepatocellular carcinoma.

### Patients

Formalin-fixed paraffin-embedded (FFPE) patient tissue samples from resected HCC were obtained from the Sun Yat-sen University Cancer Center (SYSUCC). The tissues were used for immunofluorescence (IF) assay. We collected samples from 29 patients who underwent curative resection from 2019 to 2020 with the approval of the ethics committee. Every patient consent was obtained with approval from the institutional review board (IRB) (Approval number: G2022-027-01). All the clinical details are listed in Table 1.

**Table 1:** Clinicopathological characteristics of patients

Variables	*N* (%)
Sex, male	23(82.1%)
Age, >60	7(24.14%)
TNM stage	
I	13(44.83%)
II	9(31.03%)
III	5(17.24%)
IV	2(6.90%)
Maximum tumor size (>5 cm)	9(31.03%)
Histological grade	
I	0(0.00%)
II	15(51.72%)
III	14(48.28%)
IV	0(0.00%)

### Establishment of S100 gene family associated prognostic model

The least absolute shrinkage and selection operator (LASSO) regression algorithm was used to select features for predicting prognosis in HCC using ‘glmnet’ package of R software [23]. Lasso regression algorithm could construct an optimal model, in which the loss function has an added term that allows the coefficients of a given gene to shrink to zero, thus removing that gene from the model. To improve the reliability and objectivity of the analysis results, 10-fold cross-validation was performed to identify the optimal lambda value that came from the minimum partial likelihood deviance. The risk-score formula was constructed as (eqn 1):

(1) $$\text{Risk Score}=\sum_{\text{i}=1}^{\text{N}}({\text{Exp}}_{\text{i}}\times {\text{Coe}}_{\text{i}})$$

Exp_i_ was the expression value of selected gene *i*, and the Coe_i_ was the corresponding regression coefficient.

To assess the potential clinical usefulness of the model, we performed decision curve analysis on our data [24]. Our prognostic model was compared with former risk score models in HCC based on TCGA-LIHC database. With the same probability, the clinical usefulness was better when the net benefit was greater.

### The decision curve analysis

The decision curve analysis (DCA) was performed to evaluate and compare the predictive value of our prognostic model with previous reported prediction models (Table 2). In decision curves, net benefit is taken into account, which considers both potential benefits and potential harms [25]. The horizontal axis of DCA represented the percentage of threshold probability (*P*_t_), and the vertical axis represented the net benefit of the predictive model. The net benefit was calculated using the following formula (eqn 2):

(2) $$\text{Net Benefit}=\left(\frac{\text{True positive count}}{\text{n}}\right)-\left(\frac{\text{False positive count}}{\text{n}}\right)\times \left(\frac{{\text{P}}_{\text{t}}}{1-{\text{P}}_{\text{t}}}\right)$$

**Table 2:** Prognostic models in decision curve analysis

Name	Abbreviation	Reference
Our model	Model 1	–
Jia-Yin Yang et al.’s prognostic model	Model 2	[34]
Kai Wang *et al*.’s prognostic model	Model 3	[35]

Based on this formula, true- and false-positive counts are the number of patients with true- or false positive results, and *n* is the total number of patients. The threshold probability, also called risk threshold, varied from 0 to 1.0. Therefore, the clinical usefulness was better when the net benefit was greater under the same probability.

### Tumor immune microenvironment analysis

For exploring the relationship of immune cells infiltration and risk score, the tumor immune estimation resource (TIMER) algorithm was employed to estimate the abundance of B cells, CD4^+^ T cells, CD8^+^ T cells, myeloid dendritic cells, neutrophils, and macrophages in TCGA-LIHC patients (https://cistrome.shinyapps.io/timer/, accessed on March 4, 2022) [26]. We downloaded the level of immune cell infiltration in patients with HCC and the R software pheatmap package was used to draw the correlation between different immune cells infiltration and risk score.

For estimating the association of infiltration level of specific immune cell and S100A9 expression, we used the Single-Sample Gene Set Enrichment Analysis (ssGSEA) algorithm in the R package Gene set variation analysis (GSVA) to evaluate the 24 tumor-infiltrating immune cells (TIICs) abundance [27]. GSVA is an unsupervised, non-parametric method for calculating differences in gene set enrichment over samples within an expression dataset. Each ssGSEA enrichment score reflects the extent to which the genes in a specific gene set are coordinately up- or down-regulated in a sample.

### Predictive therapeutic response

Based on the Genomics of Drug Sensitivity in Cancer (GDSC) [28], we predicted therapeutic response of 5-Fluorouracil, gemcitabine and sorafenib for HCC patient in the TCGA database. The prediction process was performed using the R package ‘pRRophetic’ [29], which was an existing algorithm for predicting clinical chemotherapeutic response based on tumor gene expression data. We further estimated the potential immune checkpoint blockage (ICB) response in these patients with the Tumor Immune Dysfunction and Exclusion (TIDE) algorithm [30].

### Analyzing heterogeneity of pathways

Proteomic data based on TCGA data were obtained from The Cancer Proteome Atlas (https://tcpaportal.org/tcpa/, accessed on March 2, 2022) [31]. The pathway scores, which were protein expression signatures of pathway activity were calculated as previously reported [32]. Pathways included were apoptosis, cell cycle, DNA damage, EMT, hormone signalling, TSC/mTOR, PI3K/Akt, Ras/MAPK, and RTK signaling. The correlation between prognostic model and pathway scores was analyzed by Spearman correlation.

### Immunofluorescence (IF) assay

Immunofluorescence (IF) assay were performed on formalin-fixed and parrffin-embedded (FFPE) tissue samples of HCC patients as described previously [13]. IF images were taken under a Leica SP8 confocal microscope (Leica Microsystems, Mannheim, Germany). Co-localization analysis was performed using JACoP plugin in Image J software. Occurrence of co-localization was calculated using Pearson’s correlation coefficient (PCC) and quantification of the co-localization was obtained using overlap coefficients (k1 and k2).

### Survival analysis

Kaplan–Meier survival analysis of the S100 family members-based risk model from the TCGA dataset was performed by log-rank test [33]. The patients were grouped into high- and low risk clusters. Overall survival curves were plotted and compared. The hazard ratio (HR) was generated by univariate cox proportional hazards regression.

### Statistical analysis

The statistical difference of the tumor and normal tissues was compared through the Wilcox test. In Kaplan–Meier curves, *P*-values and hazard ratios were generated using log-rank tests and univariate Cox proportional hazards regressions. Receiver operating characteristic (ROC) curves were used to study the prediction efficiency of the risk score model. All the analysis methods and R packages were implemented by R version 4.0.3. The *P-*value <0.05 was considered statistically significant.

## Results

### Prognostic values of S100 family members in hepatocellular carcinoma

**Figure 1:**
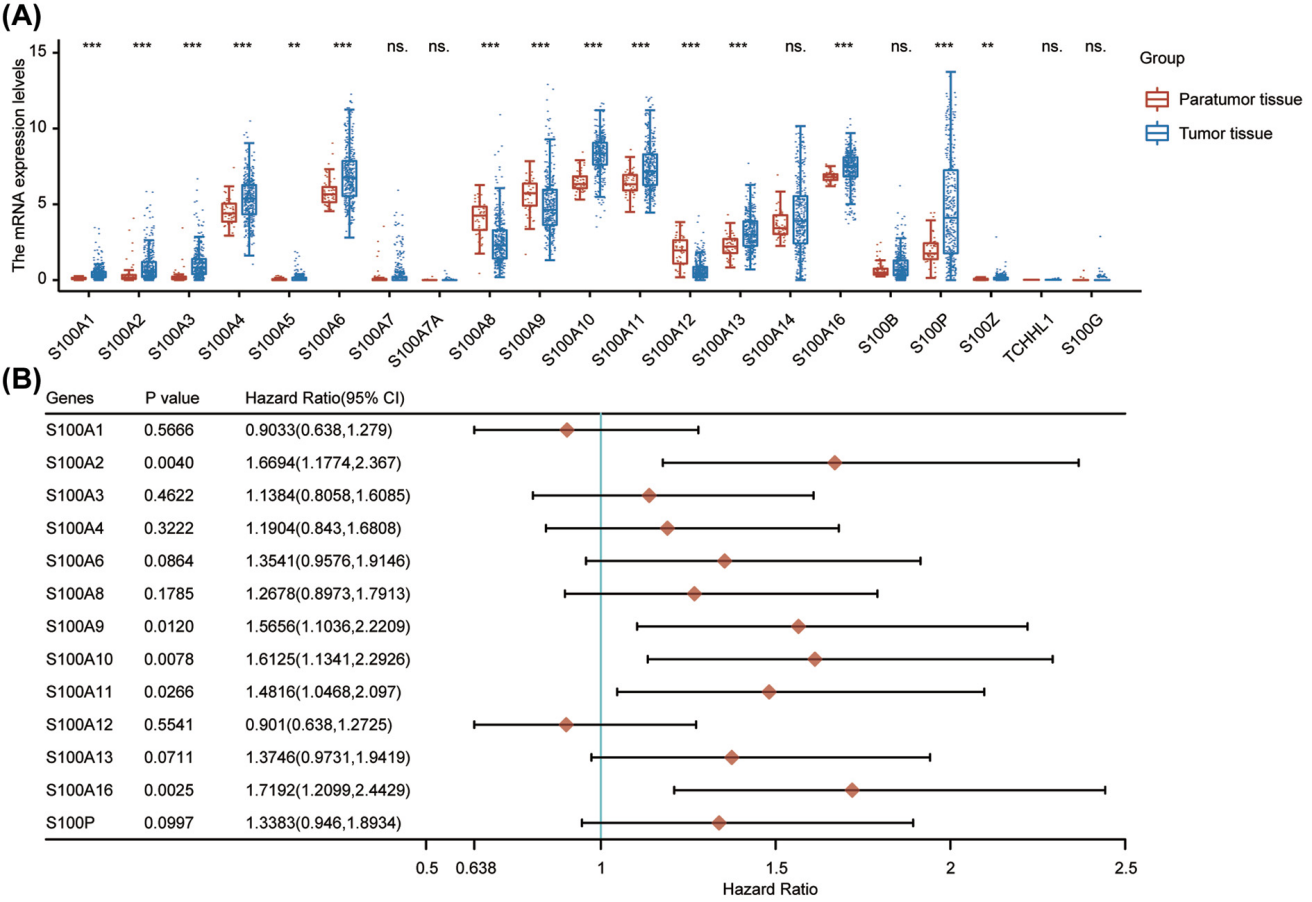
The prognostic value of S100 family members in HCC patients (**A**) The mRNA expression of individual members of S100 family in HCC. (**B**) Forest plot showed individual S100 family member associated with HCC prognosis. **P<0.01, ***P<0.001, ns., no significance. Abbreviations: CI, confidence interval; HCC, hepatocellular carcinoma.

In the first step, we evaluated the mRNA expression and prognostic significance of each S100 family member in HCC. 13 of the 21 family members were found to be highly expressed in HCC tumor samples, including S100A1, S100A2, S100A3, S100A4, S100A5, S100A6, S100A10, S100A11, S100A13, S10016, S100P, and S100Z (Figure 1A). In contrast, S100A8, S100A9, and S100A12 were highly expressed in paratumor tissues in HCC. Next, we investigated the association of differentially expressed genes and patient prognosis. As S100A5 and S100Z genes were not expressed in most HCC patients, they were not included in our following analysis. Finally, we found five genes (S100A2, S100A9, S100A10, S100A11, and S100A16) were significantly associated with overall survival (OS) of HCC patients (Figure 1B). These results indicate that the expression levels of S100 family members were significantly different between tumor and paratumor samples and associated with prognosis of HCC patients.

### Construction of S100 family members associated prognostic model

**Figure 2:**
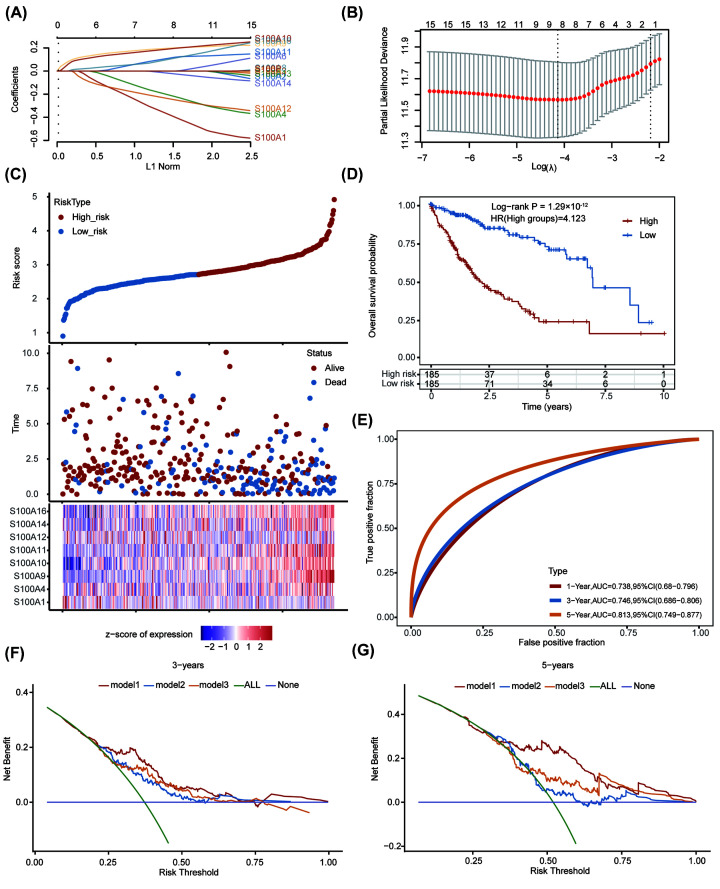
Construction of prognostic model based on S100 family genes (**A**) LASSO coefficient profiles and (**B**) partial likelihood deviance for the LASSO regression. (**C**) The relationship among the risk score, survival time and survival status of HCC patients in TCGA dataset. The upper panel showed the scatter diagram of the risk score (from low to high), and different colors represented different risk groups (blue points indicated low-risk group and red points indicated high-risk group); the middle panel showed the distribution of patient survival status and survival time, depicted as a vertical scatter plot; the bottom panel represented the expression heat map of the genes included in the prognostic model. (**D**) Kaplan–Meier curve of prognostic model. (**E**) 1-year, 3-year and 5-year ROC curves of the prognostic model. (**F** and **G**) The decision curve analysis of prognostic model with previous published prognostic models for 3-year (**F**) and 5-year OS. Abbreviations: HCC, hepatocellular carcinoma; LASSO, least absolute shrinkage and selection operator; OS, overall survival; ROC, receiver operating characteristic; TCGA, The Cancer Genome Atlas.

As S100A5, S100A7, S100A7A, S100Z, TCHHL1, and S100G are ubiquitously expressed at low levels in both tumor and paratumor samples (as shown in Figure 1A), they were not included in the following analyses. Based on the other S100 family members (S100A1, S100A2, S100A3, S100A4, S100A6, S100A8, S100A9, S100A10, S100A11, S100A12, S100A13, S100A14, S100A16, S100B, and S100P), we utilized LASSO regression analysis to develop a prognostic model for patients with HCC and finally built an 8-mRNA-based signature (S100A1, S100A4, S100A9, S100A10, S100A11, S100A12, S100A14, and S100A16) for predicting OS. LASSO regression with ten-fold cross-validation was performed to get the optimal lambda value that came from the minimum partial likelihood deviance (Figure 2A,B). A risk-score formula was developed as following (eqn 3):

(3) Risk score=(-0.4000)×S100A1+(-0.2215)×S100A4+0.2007×S100A9+0.1968×S100A10+0.1200×S100A11+(-0.2497)×S100A12+(-0.0252)×S100A14+(0.1126)×S100A16

According to this risk score model, the patients were then equally separated into low- and high-risk groups (with the median risk score used as a cut-off). The detail grouping information were shown in Supplementary Table S3. The relationship among survival status, survival time and risk score were shown in the middle panel of Figure 2C. Low-risk score samples tended to have longer survival time (Y axis) than high-risk score samples, and showed more dispersion distribution. The number of alive samples with low-risk scores was significantly higher than those with high-risk scores. The lowest panel of Figure 2C presented the heatmap of the expression of 8 S100 family members in the samples and showed that the high expression of S100A4, S100A9, S100A10, S100A11, S100A14, and S100A16 was associated with high risk, which was a risk factor. Kaplan–Meier survival analysis indicated that there was a significant difference between the two groups (Log-rank *P* = 1.29 × 10^−12^, HR = 4.123) (Figure 2D). In addition, we conducted receiver operating characteristic curve (ROC) analysis on risk score using timeROC package and analyzed prognostic classification efficiency for the 1-year, 3-year, and 5-year, respectively (Figure 2E). The results showed that the model had a high prediction performance (AUC = 0.738 for 1-year; 0.746 for 3-year; 0.813 for 5-year). In order to compare our risk score model with previous prediction models in HCC, the decision curve analysis was used (Figure 2F,G). It showed that our model (model 1) was superior to Jia-Yin Yang et al.’s prognostic model (model 2) [34] and Kai Wang et al.’s prognostic model (model 3) [35]. These results suggest that this prediction model based on part of S100 family members could be used to predict the prognosis of HCC patients, and be used as an indicator to evaluate the prognosis of patients.

### Analysis of the mutational landscape

**Figure 3:**
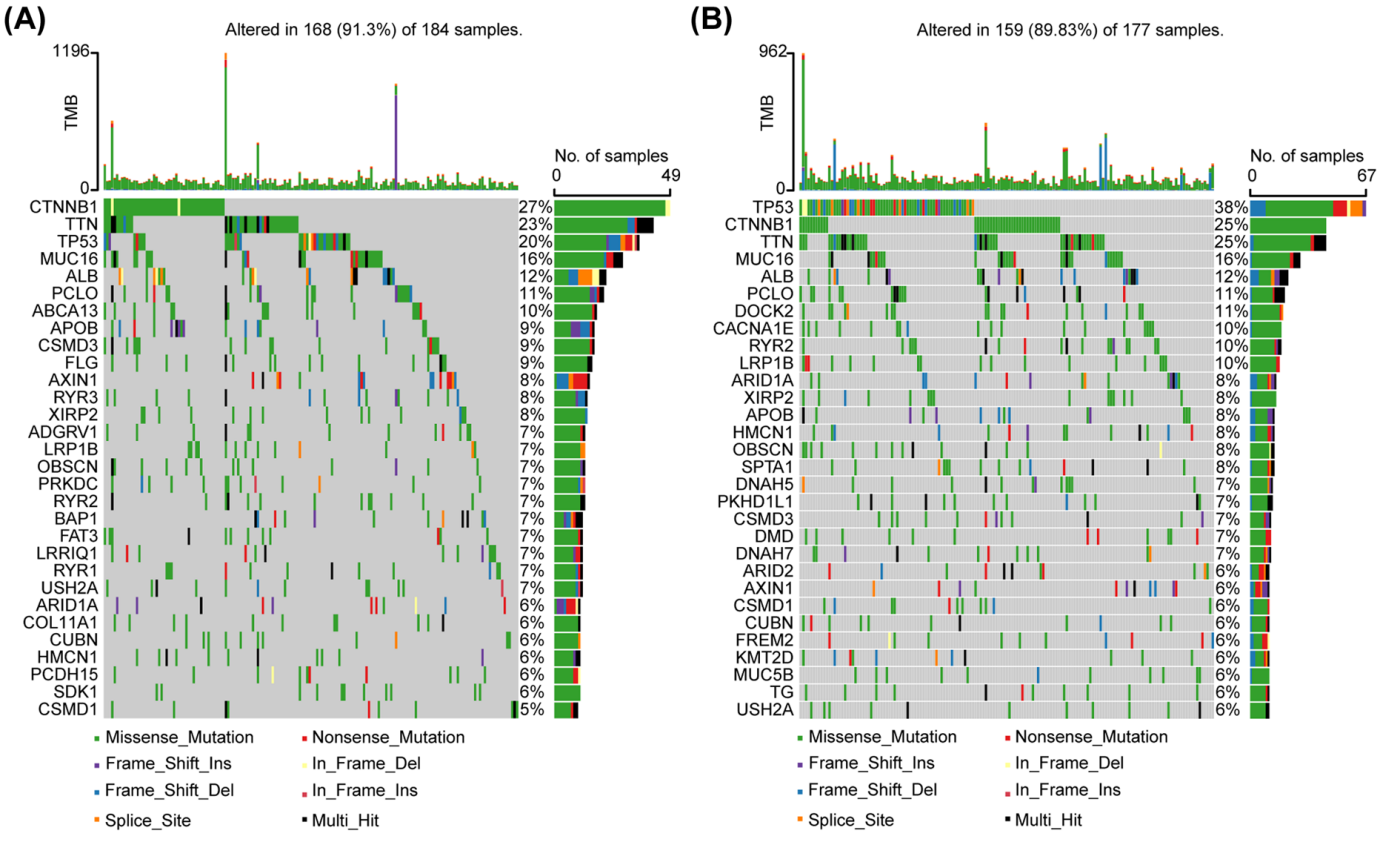
Mutational landscape of low- and high-risk groups of HCC patients in TCGA dataset (**A** and **B**) Top 30 mutational genes in individual group were shown. CTNNB1 (low-risk group) and TP53 (high-risk group) are reported as the most frequently mutated genes in separated cluster. Abbreviations: CTNNB1, cadherin-associated protein β1; No., number; TMB, tumor mutation burden; TP 53, tumor protein P53.

With the help of the ‘maftool package, we explored the landscape of somatic mutations of HCC patients in low- and high-risk groups. The top 30 common mutational genes in two groups were shown in Figure 3. Tumor protein P53 (*TP53*), titin (*TTN*), cadherin-associated protein beta 1 (*CTNNB1*), mucin 16, cell surface associated (*MUC16*), albumin (*ALB*) and piccolo presynaptic cytomatrix protein (*PCLO*) were the common top 6 frequent mutational genes in both of two groups. Low-risk group had the highest CTNNB1 mutation rate, while the high-risk group exhibited the highest TP53 mutation rate. Hiroki Murai et al. have previously classified HCC into three molecular subtypes based on transcriptomic data [36]. Class I, with the poorest prognosis, was associated with TP53 mutations, while class III with the best prognosis was associated with CTNNB1 mutations. This was consistent with the prognostic outcome in our study.

### Phenotypic heterogeneity

**Figure 4:**
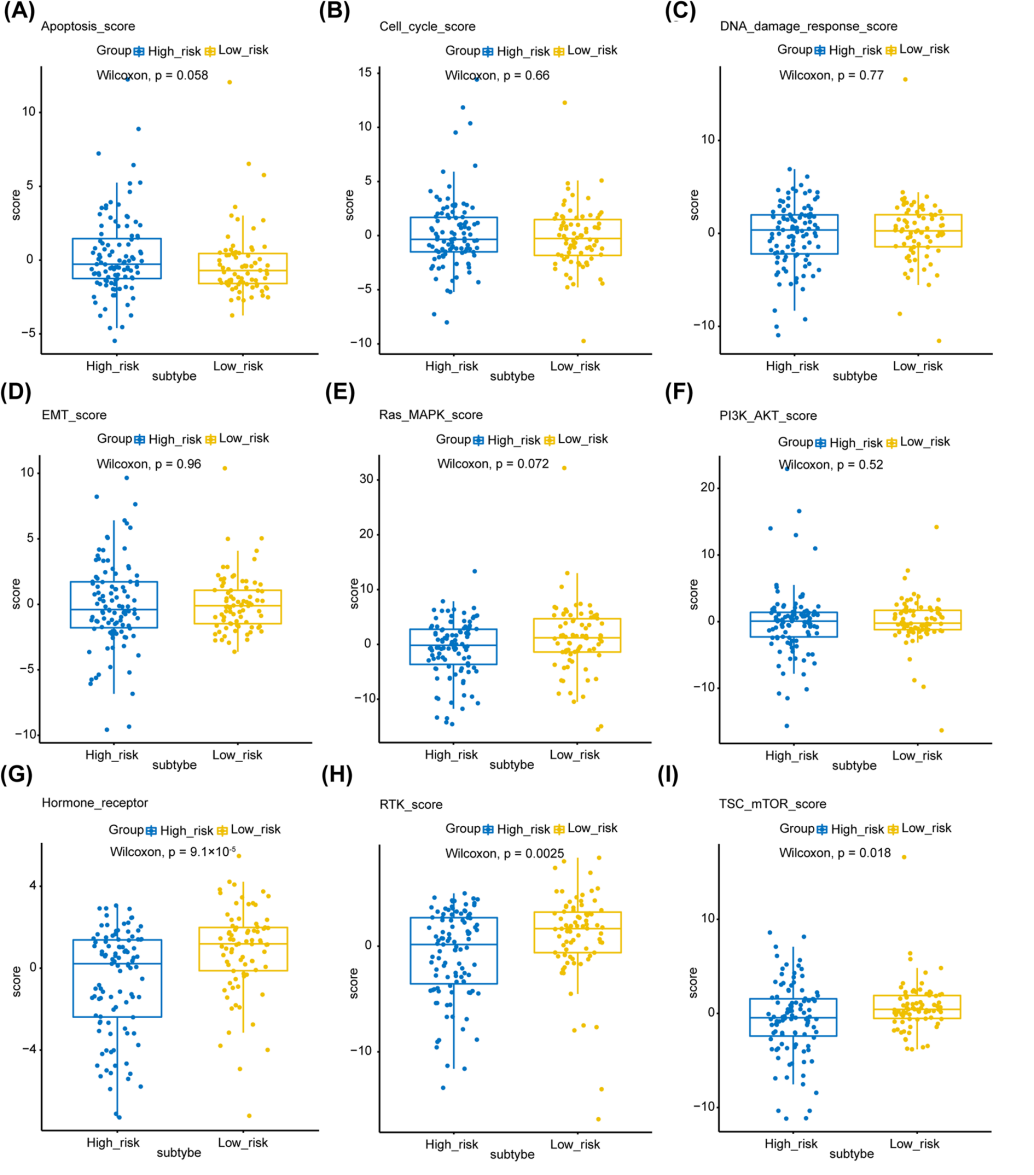
Phenotype heterogeneity among the S100 family members-based subtypes Boxplots show differences in (**A**) apoptosis, (**B**) cell cycle, (**C**) DNA damage response, (**D**) EMT, (**E**) Ras/MAPK, (**F**) PI3K/AKT, (**G**) Hormone receptor, (**H**) RTK, and (**I**) TSC-mTOR scores from TCGA among S100 family members-based subtypes. The data from A–I were from RPPA data-based scores published by TCGA. The Kruskal–Wallis test was performed to calculate the *P*-value, and those associations with *P*-value < 0.05 were considered significant. Abbreviations: DNA, deoxyribonucleic acid; EMT, epithelial–mesenchymal transition; MAPK, mitogen-activated protein kinase; mTOR, mammalian target of rapamycin; RPPA, reverse-phase protein microarray; RTK, receptor tyrosine kinase; TCGA, TCGA, The Cancer Genome Atlas; TSC, tuberous sclerosis complex.

Next, we tried to find out whether S100 family members-based subtypes in HCC showed phenotypic heterogeneity (measured as pathway scores) (Figure 4A–I). These pathway scores, which were signatures of pathway activity, were obtained from a reverse-phase protein array (RPPA) published by TCGA [32]. Our analysis showed that the pathway scores for hormone receptor, receptor tyrosine kinase (RTK), and tuberous sclerosis complex (TSC)-mammalian target of rapamycin (mTOR) were significantly higher in low-risk group. It has been reported that estrogen receptors (ERs) play an important role in regulating inflammation, iron homoeostasis, energy metabolism and other processes to protect the liver [37–39]. Furthermore, a number of studies have shown that the expression of ERα in primary HCC tissues is less than normal liver tissues or the adjacent tissues, indicating the suppressive effects of ERα in HCC [40,41]. And RTKs are recognized as key regulators of normal cellular signaling in liver and their dysfunction is associated HCC [42]. The mTOR signaling is believed to be an essential inducer for HCC development and progression, and targeting mTOR is thought to be a promising strategy for cancer therapy [43]. These results suggest that the S100 family members-based subtypes show differences in part of hepatocellular carcinoma-associated phenotypes. The inactive hormone receptor pathway may be the reason of worse outcome for patients in high-risk group.

### Predictive therapeutic response

**Figure 5:**
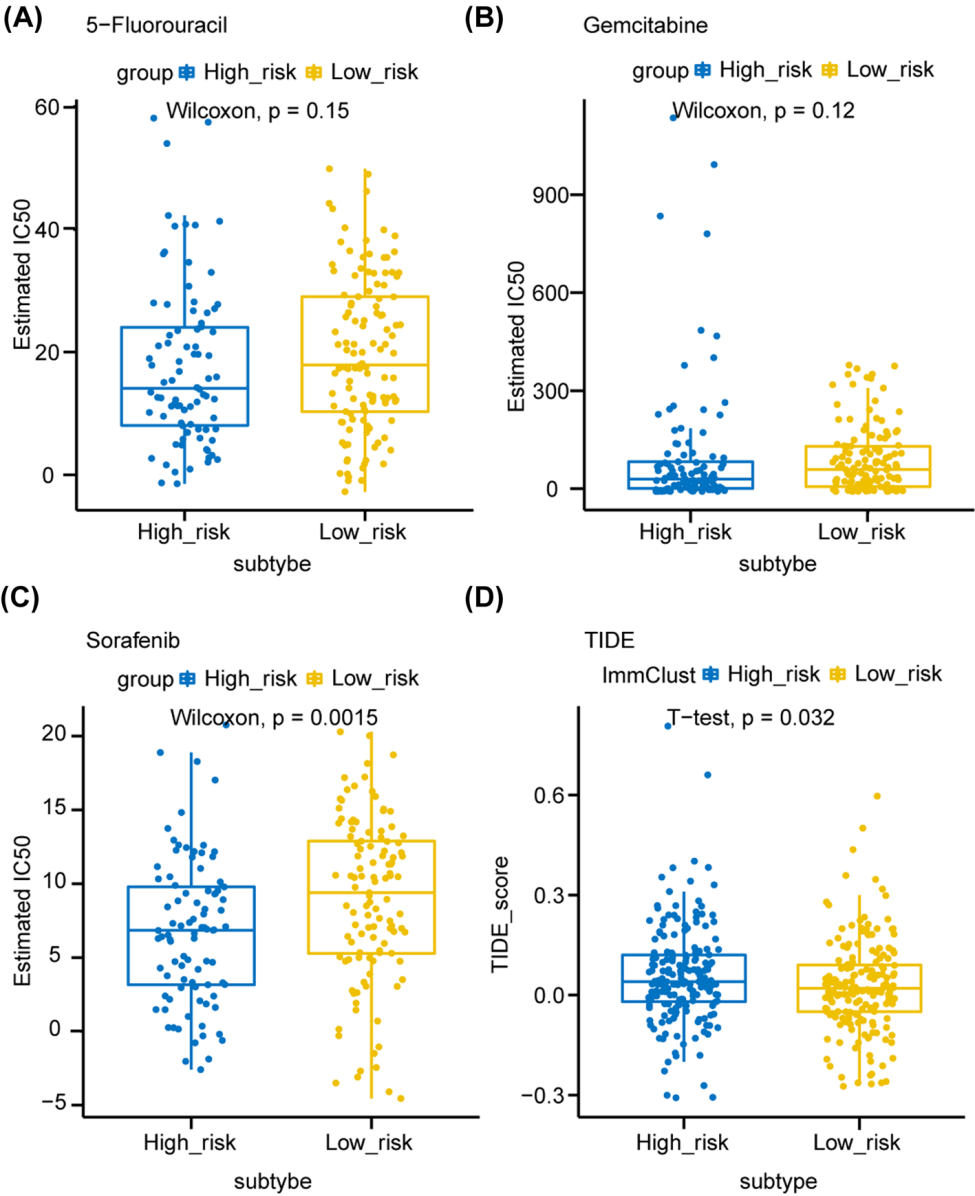
Predictive therapeutic response based on the risk score model (**A–C**) The box plots showed the estimated half maximal inhibitory concentration (IC50) for 5-fluorouracil (A), gemcitabine (B) and sorafenib among S100 family members-based subtypes. (**D**) The box plot of TIDE scores of high- and low-risk groups, indicating the predictive therapeutic response to immune checkpoint blockage therapy. Comparisons between continuous variables were performed using the Wilcoxon rank-sum test or two-sample t-test depending on normality. Abbreviation: TIDE, tumor immune dysfunction and exclusion.

We further investigated the predictive drug sensitivity among different groups based on the GDSC [28]. We found that the estimated drug sensitivity values (half maximal inhibitory concentration, IC50) of 5-fluorouracil and gemcitabine were lower in the high-risk group, indicating this group tended to be more sensitive to chemotherapy (although the differences were not statistically significant) (Figure 5A,B). Moreover, high-risk group patients were sensitive to sorafenib (*P*=0.0015), a multi-kinase inhibitor for the treatment of HCC (Figure 5C). Currently, immunotherapy-based treatment has a great role in treatment of HCC. We further explored the potential immune checkpoint blockage (ICB) response in these patients with the Tumor Immune Dysfunction and Exclusion (TIDE) algorithm (Supplementary Table S4). The TIDE scores of high-risk samples were higher than those of low-risk samples, suggesting that high-risk group was resistant to ICB therapy (Figure 5D).

### The correlation between S100 family members-based subtypes and tumor immune infiltrating cells

**Figure 6:**
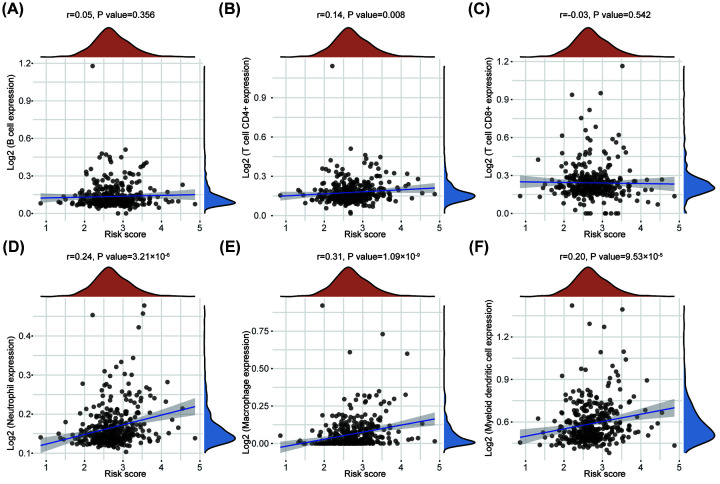
Correlation between risk score and tumor-infiltrating immune cells (**A–F**) The correlations between risk score and immune score of different kinds of immune cells was analyzed with Spearman correlation. The horizontal axis represents the distribution of the risk score, and the longitudinal axis represents the distribution of the immune score of different immune cells. The right density curve showed the distribution of the immune scores among patients. The upper density curve represented the trend in distribution of the risk score.

Based on the TCGA database, we further explored the correlation between S100 family members-based subtypes and tumor immune cells in tumor tissues using TIMER algorithm. Interestingly, macrophages exhibited the strongest correlation with S100 family members-based subtypes (*r* = 0.31), highlighting the key role of S100 family members correlated with macrophages in tumor immune infiltrating cells (Figure 6A–F).

### S100A9 is highly expressed in macrophages

**Figure 7:**
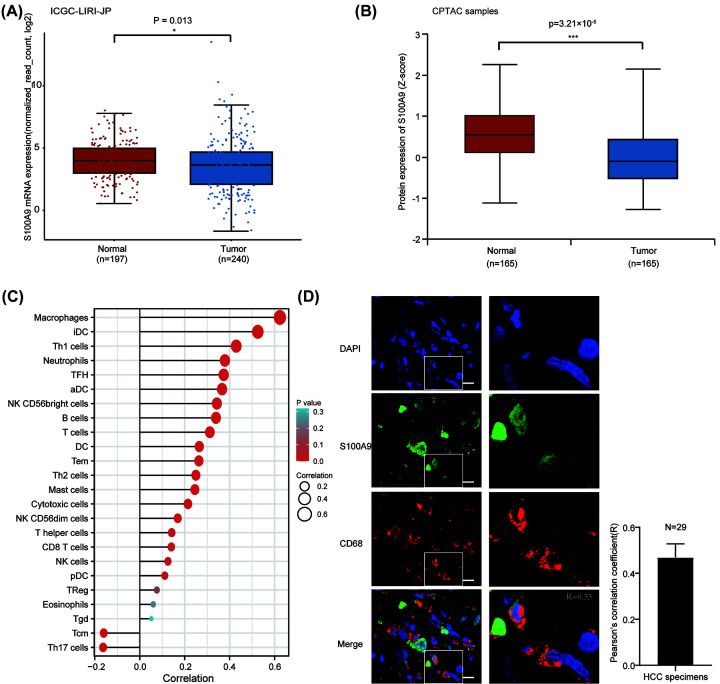
S100A9 is highly expressed in macrophages (**A** and **B**) S100A9 is predominantly expressed in normal tissues both in mRNA and protein levels. (**C**) The ssGSEA method quantifies the association of S100A9 expression with the abundance of 24 immune cells in the tumor immune microenvironment. (**D**) Co-immunofluorescent staining for S100A9 (in green) and the macrophage marker CD68 (in red) showed extensively co-localization in human HCC specimen. Pearson's correlation coefficient was calculated using JACoP tool in ImageJ. Scale bar represents 10 μm. Abbreviations: aDC, activated dendritic cell; CPTAC, Clinical Proteomic Tumor Analysis Consortium; ICGC, International Cancer Genome Consortium; iDC, immature dendritic cell; NK, natural killer cell; pDC, plasmacytoid dendritic cell; Tcm, central memory T cell; Tem, effector memory T cell; Tfh, follicular helper T cell; Tgd, gamma delta T cell; Th1, type I T helper cell; Treg, regulatory T cell.

The 8-gene prognostic model showed potential predictive value in HCC patients. As S100A9 had the highest regression coefficient in the risk score model, this indicated that it may have the greatest impact on outcome in HCC. Previous studies have demonstrated that S100A9 play an important role in promoting tumor proliferation [44], migration [45], and maintaining cancer stemness [13]. As S100A9 was highly expressed in para-tumor tissues instead of tumor tissues in HCC patients from TCGA database (Figure 1A), we suspected that it was mainly produced from cancer stromal cells. To verify this, we compared the mRNA expression of *S100A9* in tumor and normal tissues in HCC patients of the ICGC-LIRI-JP project (Figure 7A). And we confirmed that S100A9 was highly expressed in normal tissues in protein level in CPTAC cohort (Figure 7B). As immune cells were the main components in cancer stroma, we used the ssGSEA algorithm to examine the relationship between *S100A9* gene expression and tumor immune cell infiltration. The correlation between the abundance of 24 immune cells in HCC patients in TCGA-LIHC dataset and *S100A9* expression were presented in the bubble plot (Figure 7C). The results indicated that macrophages were most significantly related to the expression of the *S100A9* gene (Spearman’s correlation coefficient is 0.624, *P*<0.001). Hence, we investigated the association of macrophages and S100A9 in our HCC specimens with immunofluorescence staining, and confirmed the presence of S100A9 localized to the macrophages. These results indicate that S100A9 may be closely related with macrophages.

## Discussion

Dysregulation of S100 family members has been detected in a variety of human cancers. In this study, we explored the expression of S100 family members in HCC based on TCGA dataset. Most S100 family genes were differentially expressed between tumor and para-tumor tissues. Based on S100 family members, we constructed prognostic model using LASSO regression method, which was significant for prognostic stratification and treatment development. We identified eight S100 family genes (S100A1, S100A4, S100A9, S100A10, S100A11, S100A12, S100A14, and S100A16) that may be more important for patient prognosis.

S100A1 was up-regulated in HCC tissues and served as an oncogene by interacting with large tumor suppressor kinase 1 (LATS1) and activating yes-associated protein (YAP). It was found that large tumor size and low tumor differentiation were associated with S100A1 expression [14]. S100A4 was also known as a tumor promoting gene in HCC. Overexpression of S100A4 led to HCC metastasis through activating signal transducer and activator of transcription 3 (STAT3) phosphorylation and up-regulating osteopontin (OPN) expression [46]. The up-regulation of S100A9 was positively correlated with poor outcomes in HCC [16]. We previously reported that S100A9 could enhance HCC stemness through activating nuclear factor-kappa B (NF-κB) pathway, which may promote tumor progression [13]. The S100A10 gene was implicated in many malignancies, including thyroid carcinoma, colorectal cancer, and ovarian cancer [47]. It has been found that S100A10 expression in HCC cells was significantly higher than in normal liver cells [48]. A study by Shan et al. (2013) found that miR-590-5p inhibits HCC proliferation by down-regulating S100A10 expression [49]. As for S100A11, there is a dual role for it in the regulation of cell growth. Intracellular S100A11 inhibited cell growth, while extracellular form stimulated cell growth [50]. S100A12 was commonly expressed on neutrophils and macrophages. High expression of S100A12 was recognized as a risk factor for the overall and disease-free survival rates of HCC following curative surgical resection [51]. S100A14 was a novel member of the S100s, and had been characterized as highly expressed and functioned in HCC. High S100A14 mRNA expression correlated with poor prognosis [18]. Few studies investigated the role of S100A16 in HCC. Single-cell RNA-seq analysis indicated that S100A16 may be related with prognosis of HCC patients [52]. Therefore, these 8 S100 family genes can not only be used to develop a prognostic model to evaluate the prognosis of HCC patients but also have value for further studying the mechanism of their influence on the occurrence and development of liver cancer.

Eight S100 family genes were selected to establish the risk score model. This prognostic model was able to discriminate the high-risk cluster from the low-risk one for HCC patients in TCGA dataset. The decision curve analysis showed that our prognostic model was superior to recently reported risk score models [34,35]. This may be because that S100 family members have vital role in cellular biological processes, including Ca^2+^ homeostasis, proliferation, differentiation, apoptosis, inflammation, and cell migration and they are involved in inflammation and immune response [53]. This work highlighted the importance of S100 family members in liver cancer. However, our prognostic model does not clearly distinguish chemotherapy responses (5-fluorouracil and gemcitabine) in patients, while the model was more accurate in predicting the response to immunotherapy and targeted therapy. The one reason may be that our prognostic model was closely associated with infiltration of macrophages, which has been shown to be related with sorafenib sensitivity [54]. HCC is now generally regarded as an immunogenic tumor [55]. Numerous types of immune cells have been proved to affect the progression and prognosis of HCC. For instance, tumor-associated macrophages (TAMs) and CD4(+) CD25(+) Foxp3(+) regulatory T cells (Tregs) are associated with poor prognosis in patients with HCC [56,57]. In the present study, our results revealed that eight S100 family members played a critical role in immune infiltration of HCC. Especially, both S100A4 and S100A6 had strong positive Among these eight genes, S100A9 was the unique one that was highly expressed in paratumoral/normal tissues for HCC (Figure 7A,B). Based on ssGSEA algorithm, we validated that S100A9 was most correlated with macrophages and co-distribution of S100A9 and CD68 was found in HCC tissues. The potential function of S100A9 derived from macrophages needed to be further investigated in HCC.

However, the present study has some limitations. First, an emphasis was placed on bioinformatics analysis in the present study. The risk score model still needed to validated in external cohorts. Second, the main source of S100A9 in HCC patients should be further studied by *in vivo* or *in vitro* experiments. We were still not able to conclude that S100A9 mainly derived from macrophages. Third, our prognostic model could not be used for predicting chemotherapy response in HCC patients. In the present study, we established the new prognostic model based on S100 family members and systematically analyzed its role in discriminating different subsets of HCC patients in many aspects, including prognosis, genetic mutations, phenotypic traits, predictive therapeutic efficacy and the abundance of infiltrating immune cell. In the future, large prospective and randomized controlled studies will be needed to confirm our findings.

## Supplementary Material

Supplementary Tables S1-S4

## Data Availability

Publicly available datasets were analyzed in the present study. The data can be found here: TCGA database (https://portal.gdc.cancer.gov); GEO database (http://www.ncbi.nlm.nih.gov/geo/); ICGC database(https://dcc.icgc.org/); CPTAC database (https://proteomics.cancer.gov/programs/cptac).

## Abbreviations

ALB: albumin
CPTAC: Clinical Proteomic Tumor Analysis Consortium
CTNNB1: cadherin-associated protein beta 1
EMT: epithelial–mesenchymal transition
ER: estrogen receptor
FFPE: formalin-fixed paraffin-embedded
GDC: Genomic Data Commons
GDSC: Genomics of Drug Sensitivity in Cancer
HCC: hepatocellular carcinoma
ICB: immune checkpoint blockage
IF: immunofluorescence
IRB: institutional review board
LASSO: the least absolute shrinkage and selection operator
LATS1: large tumor suppressor kinase 1
MAPK: mitogen-activated protein kinase
mTOR: mammalian target of rapamycin
MUC16: mucin 16, cell surface associated
NF-κB: nuclear factor-kappa B
OS: overall survival
PCLO: piccolo presynaptic cytomatrix protein
ROC: receiver operating characteristic
RPPA: reverse-phase protein array
RTK: receptor tyrosine kinase
ssGSEA: Single-Sample Gene Set Enrichment Analysis
S100s: S100 protein family members
STAT3: signal transducer and activator of transcription 3
TCGA: the cancer genome atlas
TIDE: Tumor Immune Dysfunction and Exclusion
TIICs: tumor-infiltrating immune cells
TIMER: the tumor immune estimation resource
TP53: tumor protein P53
TSC: tuberous sclerosis complex
TTN: titin
